# Diagnostic spirometry in COPD is increasing, a comparison of two Swedish cohorts

**DOI:** 10.1038/s41533-023-00345-8

**Published:** 2023-06-02

**Authors:** Åsa Athlin, Karin Lisspers, Mikael Hasselgren, Björn Ställberg, Christer Janson, Scott Montgomery, Maaike Giezeman, Marta Kisiel, Anna Nager, Hanna Sandelowsky, Mats Arne, Josefin Sundh

**Affiliations:** 1 School of Medical Sciences, Faculty of Medicine and Health, Örebro University, Örebro, Sweden; 2 Department of Public Health and Caring Sciences, Family Medicine and Preventive Medicine, Uppsala University, Uppsala, Sweden; 3 Centre for Clinical Research and Education, Region Värmland, Karlstad, Sweden; 4 Department of Medical Sciences, Respiratory, Allergy and Sleep Research, Uppsala University, Uppsala, Sweden; 5 Clinical Epidemiology and Biostatistics, School of Medical Sciences, Örebro University, Örebro, Sweden; 6 Clinical Epidemiology Division, Department of Medicine, Solna, Karolinska Institutet, Stockholm, Sweden; 7 Department of Epidemiology and Public Health, University College London, London, UK; 8 Department of Medical Sciences, Occupational and Environment Medicine, Uppsala University, Uppsala, Sweden; 9 Division of Family Medicine and Primary Care, Inst NVS, Karolinska Institutet, Stockholm, Sweden; 10 Academic Primary Health Care Centre, Region Stockholm, Stockholm, Sweden; 11 Department of Respiratory Medicine, School of Medical Sciences, Faculty of Medicine and Health, Örebro University, Örebro, Sweden

**Keywords:** Outcomes research, Chronic obstructive pulmonary disease, Diagnosis

## Abstract

Spirometry should be used to confirm a diagnosis of chronic obstructive pulmonary disease (COPD). This test is not always performed, leading to possible misdiagnosis. We investigated whether the proportion of patients with diagnostic spirometry has increased over time as well as factors associated with omitted or incorrectly interpreted spirometry. Data from medical reviews and a questionnaire from primary and secondary care patients with a doctors’ diagnosis of COPD between 2004 and 2010 were collected. Data were compared with a COPD cohort diagnosed between 2000 and 2003. Among 703 patients with a first diagnosis of COPD between 2004 and 2010, 88% had a diagnostic spirometry, compared with 59% (*p* < 0.001) in the previous cohort. Factors associated with not having diagnostic spirometry were current smoking (OR 2.21; 95% CI 1.36–3.60), low educational level (OR 1.81; 1.09–3.02) and management in primary care (OR 2.28; 1.02–5.14). The correct interpretation of spirometry results increased (75% vs 82%; *p* = 0.010). Among patients with a repeated spirometry, 94% had a persistent FEV_1_/FVC or FEV_1_/VC ratio <0.70.

## Introduction

Chronic obstructive pulmonary disease (COPD) should be considered in patients who present with respiratory symptoms such as dyspnoea, cough, sputum production and a history of risk factors, especially smoking^1,2^. Spirometry is required to confirm a chronic obstruction and to confirm the diagnosis, using the ratio of the post-bronchodilator forced expiratory volume in one second (FEV_1_) and the forced vital capacity (FVC) to less than 0.70^1,3^. However, several studies from various countries show a substantial underuse of spirometry in the initial assessment of patients with suspected COPD^2–7^. Both over- and underdiagnosis of COPD may thus occur, leading to suboptimal COPD care^8–10^. In Sweden, COPD is mainly diagnosed and managed in primary care^11^, where access to spirometers has increased during the last decades^12^.

In 2005, the first Swedish PRAXIS study COPD cohort with patients from both primary and secondary care was created (PRAXIS I). In this cohort, 59% of patients had a spirometry performed at diagnosis of COPD^13^.

In 2014, a new cohort of COPD patients (PRAXIS II) was recruited from the same geographic area. During the study period, the Swedish Board of Health and Welfare and the Swedish Medical Agency published guidelines for the diagnosis and management of patients with COPD based on the Global initiative for chronic Obstructive Lung Disease (GOLD) recommendations^14–16^.

The primary aim of this study was to investigate whether the proportion of performed spirometries at COPD diagnosis increased after the new national guidelines were implemented, and to investigate patient-related factors associated with a COPD diagnosis without a diagnostic spirometry. Secondary aims were to assess whether spirometries were correctly interpreted regarding COPD diagnosis, defined as airway obstruction FEV_1_/FVC < 0.70, and whether the obstruction was persistent at follow-up.

## Results

### Patient characteristics

Of the 703 patients with a new diagnosis of COPD during 2004–2010, 375 (53%) were women and 507 (82%) were assessed in primary care (Table 1). Diagnostic spirometry was performed for 619 (88%) patients. Of these, 567 (92%) had assessable data and 102 of the 567 had FEV_1_/FVC or FEV_1_/VC ≥ 0.70. Where data was assessable for categorisation into GOLD stages 1–4 based on FEV_1_ in the percentage of predicted (FEV_1_%pred) (*n* = 532), 141 patients (25%) were categorised as stage 1 and 284 (50%), 94 (17%) and 13 (2%) into stages 2, 3 and 4, respectively.

**Table 1:** Patient characteristics distributed over performed and not performed diagnostic spirometry.

	Spirometry performed *N* (%) *n* = 619	Spirometry not performed *N* (%) *n* = 84	*p* value
Sex			0.329
Female	326 (53)	49 (58)	
Male	293 (47)	35 (42)	
Level of care			0.025
Primary	507 (82)	77 (92)	
Secondary	112 (18)	7 (8)	
Age			0.127
<65	197 (32)	22 (26)	
65–69	177 (29)	19 (23)	
≥70	245 (40)	43 (51)	
Current smoker^a^			0.001
Yes	164 (27)	37 (45)	
No	440 (73)	46 (55)	
BMI^b^			0.346
<20	46 (8)	6 (8)	
20–24.9	195 (32)	29 (36)	
25–29.9	224 (37)	22 (28)	
≥30	136 (23)	23 (29)	
CAT^c^			0.220
<10	193 (33)	23 (30)	
10–19	238 (40)	39 (50)	
≥20	162 (27)	16 (21)	
Exacerbations in recent 12 months^d^			0.491
0	485 (80)	67 (80)	
1	78 (13)	8 (10)	
2	18 (3)	4 (5)	
≥3	22 (4)	5 (6)	
Level of education			
Low	337 (54)	59 (70)	0.006
High	282 (46)	25 (30)	
Diabetes	103 (17)	16 (19)	0.581
Hypertension	315 (51)	46 (55)	0.505
Depression	128 (21)	18 (21)	0.874
Ischemic heart disease	93 (15)	14 (17)	0.694
Chronic heart failure	49 (8)	11 (13)	0.111
Comorbid asthma	59 (10)	11 (13)	0.306

*BMI* Body Mass Index, *CAT* COPD Assessment Test. Missing data: ^a^smoking, *n* = 16, ^b^BMI, *n* = 22, ^c^CAT, *n* = 32, ^d^exacerbations, *n* = 16.

Patients without a diagnostic spirometry were significantly more often from primary care sites, had a lower educational level and were more often current smokers than those with a diagnostic spirometry (Table 1). These associations remained in the multivariate logistic regression (Table 2).

**Table 2:** Factors associated with not having performed diagnostic spirometry, *n* = 84.

	OR (95% CI) Unadjusted	*p* value	OR (95% CI) Adjusted	*p* value
Female sex	1.26 (0.79–2.00)	0.33	1.21 (0.75–1.95)	0.44
Age (years)				
<65	Ref		Ref	
65–69	0,96 (0.50–1.84)	0.91	1.01 (0.52–1.95)	0.98
≥70	1.57 (0.91–2.72)	0.11	1.62 (0.91–2.89)	0.10
Current smoker	2.16 (1.35–3.45)	0.001	2.21 (1.36–3.60)	0.004
Low educational level	1.98 (1.20–3.24)	0.007	1.81(1.09–3.02)	0.022
Primary care	2.43 (1.09–5.41)	0.030	2.28 (1.02–5.14)	0.046

Multivariate logistic regression with the factor “no diagnostic spirometry” as dependent variable. Adjusted for age, sex and patient characteristics significant in univariate logistic regression. *OR* Odds Ratio.

Patients with a registered COPD diagnosis yet an FEV_1_/FVC ratio ≥0.70, i.e. by definition an “incorrectly interpreted spirometry”, were independently more often women, had hypertension, diabetes, milder COPD or a change of diagnosis from COPD to asthma during the study period when compared with those with an FEV_1_/FVC ratio <0.70 (Table 3).

**Table 3:** Factors associated with an FEV_1_/FVC or FEV_1_/VC ratio ≥ 0.70, *n* = 102.

	OR (95% CI) Unadjusted	*p* value	OR (95% CI) Adjusted	*p* value
Female sex	1.60 (1.03–2.47)	0.04	8.54 (3.76 to 19.4)	<0.001
Age				
≤64	Ref		Ref	
65–69	0.82 (0.48–1.39)	0.45	0.89 (0.47–1.68)	0.72
≥70	0.61 (0.37–1.02)	0.06	0.59 (0.32–1.10)	0.10
Primary care	3.35 (1.50–7.46)	0.003	2.56 (0.99–6.66)	0.05
Hypertension	1.67 (1.07–2.58)	0.02	2.02 (1.17–3.48)	0.01
Diabetes	2.40 (1.45–3.97)	<0.001	2.13 (1.16–3.90)	0.015
Changed diagnosis from COPD to asthma	7.11 (2.64–19.2)	<0.001	9.93 (3.10-–31.8)	<0.001
Lung function categories^a^				
FEV_1_%pred ≥80	19.4 (4.55–82.4)	<0.001	87.8 (17.0–454.4)	<0.001
FEV_1_%pred 50–79	12.6 (3.02–52.7)	<0.001	15.2 (3.56–65.0)	0.001
FEV_1_%pred ≤49	Ref		Ref	

Multivariate logistic regression with the ratio of FEV_1_/FVC or FEV_1_/VC ≥ 0.70 as dependent variable. Adjusted for age, sex and patient characteristics significant in univariate logistic regression. *FEV*_*1*_ forced expiratory volume in 1 second, *FVC* forced vital capacity, *FEV*_*1*_*%pred* forced expiratory volume in 1 second as percentage of predicted value, *OR* Odds Ratio. Missing data: ^a^Lung function categories, *n* = 35.

### Comparison of the two cohorts

**Fig. 1:**
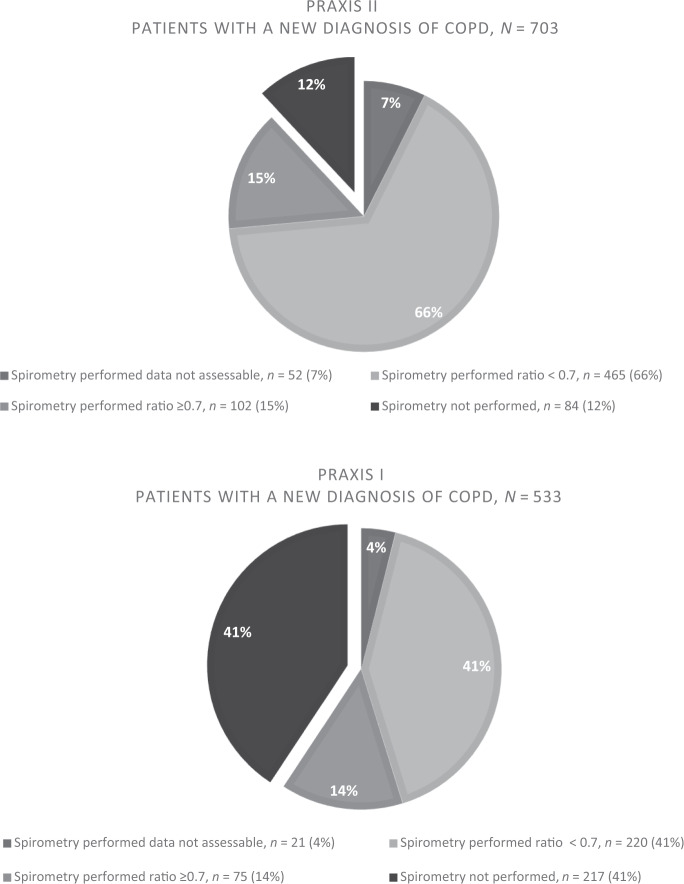
Diagnostic spirometry in patients with a new diagnosis of COPD. Comparison of COPD cohorts PRAXIS I, patients diagnosed in the period 2000–2003 (below) and PRAXIS II, patients diagnosed in the period 2004–2010 (above).

The proportion of diagnostic spirometries increased from 59% to 88% (*p* < 0.001) when comparing the two cohorts, PRAXIS I and PRAXIS II (Fig. 1).

**Fig. 2:**
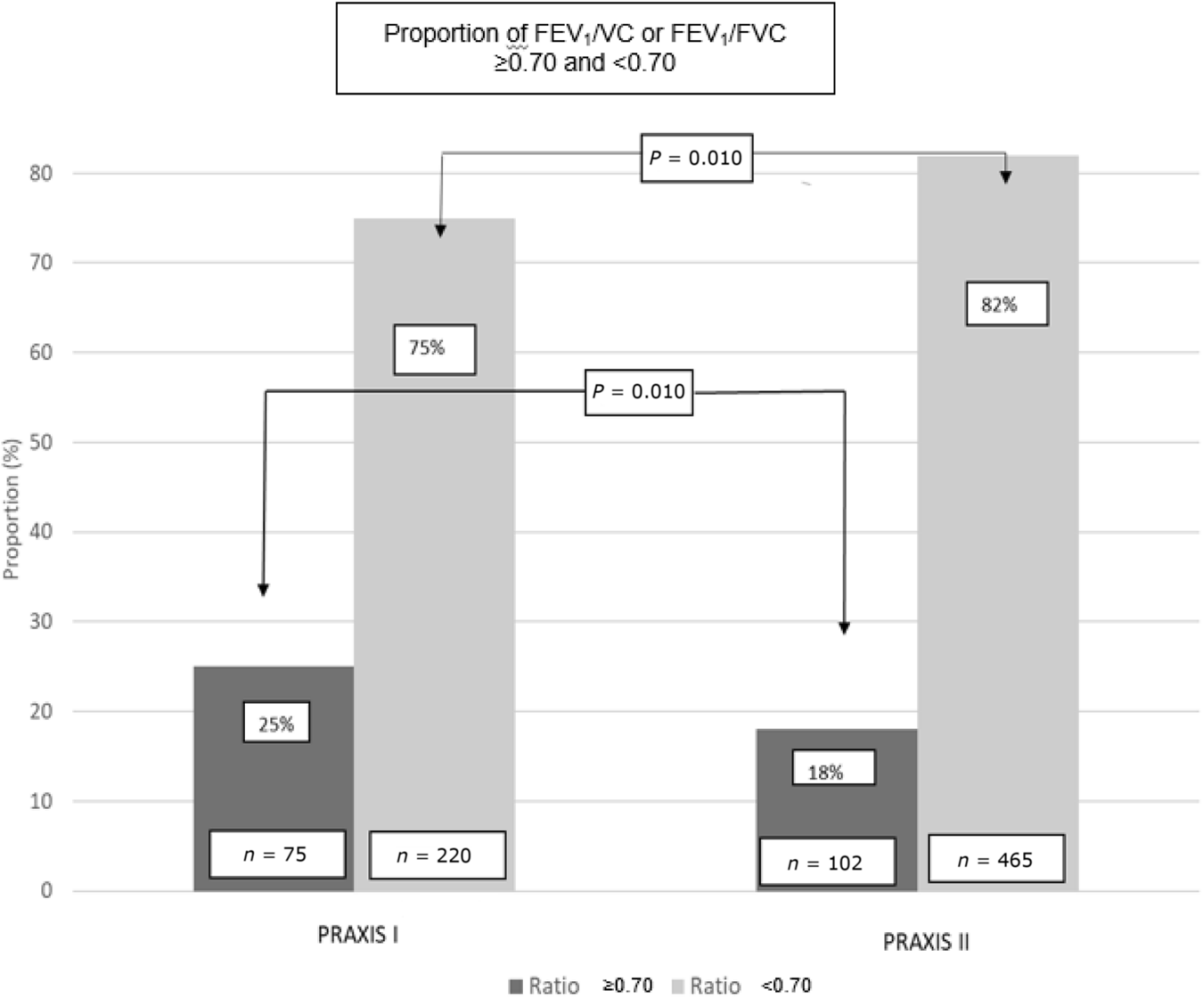
Proportion of patients with spirometry result FEV_1_/FVC or FEV_1_/VC above/equal to or under 0.70. Patients with assessable spirometry data and performed diagnostic spirometries from COPD cohorts PRAXIS I (*n* = 295) and PRAXIS II (*n* = 567).

Additionally, the proportion of correctly interpreted spirometry results in patients with assessable spirometry data increased significantly over time (75% vs. 82%; *p* = 0.010) (Fig. 2).

### Follow-up spirometry

Of the 465 patients with a correct diagnostic FEV_1_/FVC or FEV_1_/VC ratio at the time of diagnosis, 355 (76%) underwent a follow-up spirometry (mean follow-up time 52.8 months (SD 29.0)). Of these, 334 (94%) had a persistent FEV_1_/FVC or FEV_1_/VC ratio <0.70.

## Discussion

The primary finding of our study was that in patients with a doctors’ diagnosis of COPD, spirometry performed within six months of diagnosis increased from 59% in the first PRAXIS cohort of 2000–2003 to 88% in the second PRAXIS cohort of 2004–2010. Factors associated with having a COPD diagnosis without a diagnostic spirometry were current smoking, low educational level and being managed in primary care. Secondary findings were that the FEV_1_/FVC or FEV_1_/VC ratios were incorrectly interpreted regarding COPD diagnosis in 18% of the cases. This finding was more likely in females, patients with concomitant hypertension or diabetes and those who were managed in primary care. In 94% of the patients with a correct COPD diagnosis, and where a follow-up spirometry was available in the records, airway obstruction was persistent over time.

Previously reported results from the first PRAXIS cohort^14^ are consistent with other studies reporting that diagnostic spirometry was performed in about half to two-thirds of patients with a COPD diagnosis^4,5^. However, the present study from the second PRAXIS cohort shows that the frequency of performed diagnostic spirometries has clearly increased over time. There was also an increase in the proportion of correctly interpreted spirometries. We find it encouraging that the management of COPD in Sweden has improved and now complies with international and national diagnostic guidelines to a higher degree. The most important independent factor associated with not having performed a diagnostic spirometry was current smoking. We speculate that the clinical diagnosis of COPD in current smokers with respiratory symptoms may seem more obvious to physicians and thus explain the lower degree of confirmation with spirometry. Our finding is in line with a qualitative study by Joo et al. that presented a similar explanation of physicians´ motives towards diagnosis of COPD^17^. Feng et al. conclude that people with multiple unhealthy lifestyles, including smoking, are less prone to consult primary health care^18^. Consequently, they would therefore not be referred to secondary care. This could explain the difference in primary and secondary care concerning the proportion of patients that did not undergo a diagnostic spirometry.

Smoking, poverty and low education are important factors associated with a higher burden of disease^19^. This is in line with our result of low education being associated with not having performed a spirometry. Low socioeconomic status can be a reason why patients refrain from seeking care^20^. In Sweden, however, healthcare is financed with taxes and is equally available for everyone, thus the financial cost of healthcare cannot explain the lower frequency of spirometry testing in patients with low education. We speculate that our results could in part be due to a different care-seeking behaviour. For instance, smoking is found to be associated with reduced likelihood of care-seeking^21^. This highlights the importance of using a holistic approach and being aware of health inequalities when managing potential COPD patients, in order for individualised care and effective smoking cessation to be delivered. We believe that this result follows a pattern evident in other studies that have demonstrated associations between higher education and a higher degree of adherence to smoking cessation interventions and greater interest in learning self-management skills^22,23^.

The proportion of spirometries not consistent with a correct diagnosis of COPD was 18%. Similar misdiagnosis of COPD has been described previously, yet to a larger extent than in our study population^7,24^. In this particular group, we found a significant change of diagnosis from COPD to asthma over the study period (OR 9.90, 95% CI 3.09–31.78), indicating that these patients may have had asthma and not COPD from the beginning. Distinguishing asthma from COPD may be difficult, as untreated asthma may also have a temporary or persistent airway obstruction. The complexity of this differentiation was recently shown in a large global study^25^. We thus believe that some of the patients with a COPD diagnosis in our study may have had a suboptimally treated asthma where the diagnosis was changed from COPD to asthma after treatment and follow-up. Patients could also have had both asthma and COPD. A post hoc analysis showed 8.8% of patients with FEV_1_/FVC ≥ 0.70 had both a diagnosis of asthma and COPD recorded. In the group with FEV_1_/FVC < 0.70 the corresponding number was 9.6%, a non-significant difference. Another potential explanation of a COPD diagnosis in spite of a normal ratio is presence of “preserved ratio impaired spirometry” (PRISm), which can increase the risk of COPD, as well as nonpulmonary conditions in the future^26^. In a post hoc analysis, some 60% (*n* = 61) of the patients with an incorrect COPD diagnosis actually had PRISm. This may have contributed to a clinical diagnosis of COPD. Of these, 43 patients had overweight or obesity where the restrictive impairment of high BMI could have masked obstruction^26^. Furthermore, 6% of the patients did not have a persistent airflow obstruction when the first spirometries were compared to later ones. This may indicate an initial misdiagnosis, as these patients could have had asthma instead. This finding is consistent with a large UK study in which patients with an established COPD diagnosis did not have persistent airflow obstruction in 11.5% of cases^27^. Aaron et al. conclude that a single spirometric assessment may not be reliable for diagnosing patients with COPD, especially in patients with spirometry results close to the FEV_1_/FVC threshold^28^. An important clinical implication of our and others´ findings is that spirometry should be repeated after treatment has been initiated in cases of newly diagnosed COPD.

A COPD diagnosis despite an FEV_1_/FVC or FEV_1_/VC ≥ 0.70 was more common in patients who had concomitant hypertension and diabetes. We speculate that this may indicate that the focus of the consultation was on these conditions rather than COPD. On the other hand, a post hoc analysis adding a merged index of all comorbid conditions listed in Table 1 to the multivariable model did not change the results significantly (data not shown).

We find it reasonable that the number of performed spirometries and the proportion of correct interpretations is higher in specialised pulmonology care than in primary care, and that the identification of airway obstruction is easier when COPD is more severe.

We believe the increase in the proportions of performed and correctly interpreted spirometries over time is a result of an extensive national educational effort to update and implement recommendations on the assessment of COPD. Furthermore, access to spirometers in primary care in Sweden is high. At the sites of our PRAXIS II cohort, 98% of the participating healthcare units reported that they had access to spirometers. Although the proportion of performed spirometries has increased, there is still room for improvement. In Denmark, general practitioners who participated in an educational programme showed substantial improvement in the assessment of patients with COPD^29^. This is in conformity with Sandelowsky et al. who concluded that educational interventions enhanced knowledge of COPD management in primary care in Sweden^30^. In Finland, a national programme for COPD prevention and treatment had significant positive consequences, including an increase in the use of spirometry^31,32^. Since most patients with COPD are diagnosed and managed in primary care, we believe continuous education addressed to primary care physicians is of great importance.

A finding related to the interpretation of spirometries was that female sex was significantly associated with a COPD diagnosis despite a ratio ≥0.70. The reason for this is unclear. A potential explanation could have been that FEV_1_/FVC or FEV_1_/VC were closer to 0.70 for women. However, a post hoc analysis showed that ratios did not differ significantly between sexes (data not shown). Our result is in contradiction to a Spanish study where women were found not to have a COPD diagnosis despite fulfilled spirometric COPD criteria^33^. We speculate that, as symptoms of other diseases can have different clinical manifestations in women than in men^34,35^, the higher degree of spirometries with a ratio ≥ 0.70 in women with a diagnosis of COPD may mirror a more difficult differential diagnostic situation.

A major strength of our study is that it is a real-world study with a large sample size. This contributes to a high external validity and generalisability. Another strength is that, to the best of our knowledge, there are few studies with a follow-up of diagnostic assessment in two different cohorts from the same geographic area^28^. Limitations include the changes in national recommendations using FEV_1_/FVC instead of FEV_1_/maximum VC to confirm the diagnosis of COPD during the study period and that post-bronchodilator values were not present in all patients. Further, spirometry data was not always interpretable, which means a loss of patient data. However, the number of non-assessable spirometries was very low, and an attrition analysis showed that patients included in the analysis of spirometry interpretation and those who were excluded due to non-assessable spirometry data did not differ between any of the variables presented in Table 1 (data not shown). When completing a questionnaire there could be a risk of recall bias.

The second PRAXIS cohort included more primary health care centres (PHCCs) than the first PRAXIS cohort. However, a random selection was performed in both cohorts. Additional analysis where the extra PHCCs were excluded showed substantially unchanged results (data not shown).

The use of spirometry to confirm COPD diagnosis has increased over time, indicating improved implementation of COPD guidelines. At risk of not undergoing a diagnostic spirometry were current smokers, patients with low education and those managed in primary care. There is still a need for continuous medical educational activities to increase diagnostic accuracy.

## Methods

### Design and study population

The PRAXIS study is a real-life observational cohort study including primary and secondary care patients with a doctor’s diagnosis of asthma and COPD, respectively, in central Sweden. The COPD part of the project includes two different cohorts with randomly selected patients with COPD. The first PRAXIS cohort (PRAXIS I) was assessed using record reviews of the years 2000 to 2003 and a questionnaire from 2005. The second PRAXIS cohort (PRAXIS II) includes patients with record reviews from 2004 to 2014 and a questionnaire from 2014.

**Fig. 3:**
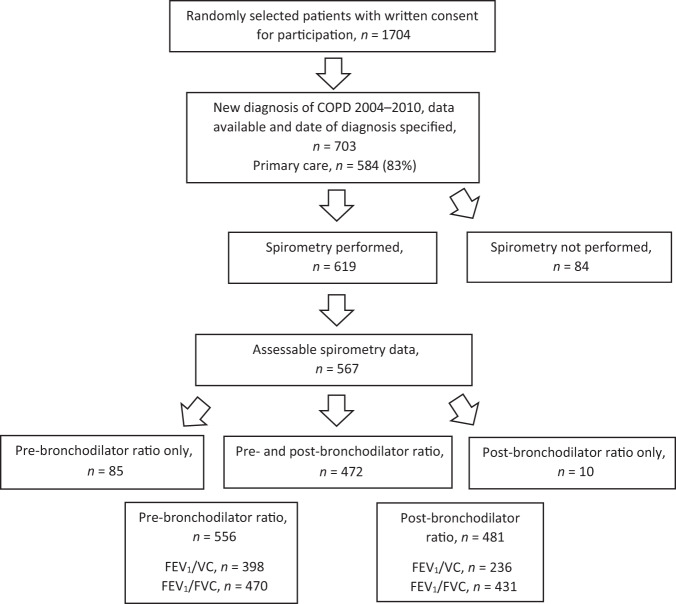
Patient selection – PRAXIS II cohort. Patients with a new diagnosis of COPD and performed/not performed diagnostic spirometry.

In the present study, patients were sampled from PRAXIS II. Patients were enrolled from the central hospitals, seven randomly selected district hospitals and 76 randomly selected PHCCs in seven regions in central Sweden. At each centre, all patients aged 18–75 years with a doctor’s diagnosis of COPD (ICD-10 code J44) in their medical records during the period 2007–2010 were listed. An internet-based random selection (random.org) was performed from each site, resulting in a study basis of 2310 patients. In 2014 a letter of consent together with a questionnaire was sent to a total of 2310 patients, of which 1704 (74%) agreed to participate and returned the completed questionnaire. A review of the medical records was performed. This identified the present study population of 703 patients, 584 (83%) from primary and 119 (17%) from secondary care, who had received a diagnosis of COPD for the first time between 2004 and 2010 (Fig. 3). Previously published spirometry data from PRAXIS I was used to enable a comparison over time^14^.

### Data collection and variables in the study

The questionnaire included sociodemographic data and information on health status including:

- Age categorised as <65, 65–69 and ≥70 years. For the main analysis, age at the time of returning the questionnaire was used. For the presentation of spirometry staging according to GOLD, age at performed spirometry was used.
- Educational level: low educational level defined as <2 years beyond the nine years of Swedish compulsory school, and high educational level as ≥2 years beyond compulsory school.
- Body Mass Index (BMI): underweight categorised as <20, normal weight as 20–24.9, overweight as 25–29.9 and obesity as ≥30 kg/m^2^.
- Smoking status categorised as current daily smoking or not.
- Exacerbations defined as a deterioration of the disease that required a course of antibiotics and/or oral steroids and/or an emergency visit and were presented as 0, 1, 2 and >2 during the previous 12 months.
- Health status assessed by the Swedish version of the COPD Assessment Test (CAT)^12^. The CAT scores were categorised into low, medium and high ( < 10, 10–19 and ≥20, respectively), according to GOLD^3^.

Data on spirometry and comorbid conditions were retrieved by review of medical records for the period 2004–2014. Comorbidities were retrieved by diagnostic codes. Diabetes was defined as a diagnosis of types 1 or 2 diabetes mellitus and depression as a recorded diagnosis with or without concomitant antidepressant drug treatment.

### Spirometry

Diagnostic spirometry was defined as a spirometry performed during the interval starting six months prior to diagnosis of COPD and ending six months after the first date of diagnosis. Spirometry data were available either as a separate spirometry report or as a part of the medical record.

For assessment of whether the interpretation of the diagnostic spirometry was correct, data on all available pre- and post-bronchodilator values and ratios from FEV_1_, FVC and VC were collected. A ratio of FEV_1_/FVC or FEV_1_/VC < 0.70 was considered a “correctly interpreted spirometry” and a ratio of FEV_1_/FVC or FEV_1_/VC ≥ 0.70 was considered as an “incorrectly interpreted spirometry”.

According to current guidelines, a post-bronchodilator FVC should be performed at diagnostic spirometries^1^. Earlier guidelines in Sweden recommended a post-bronchodilator VC. Depending on which data was available we chose to use either FEV_1_/FVC or FEV_1_/VC to assess whether the COPD diagnosis was correct. If both pre- and post-bronchodilator values were available, only post-bronchodilator values were used. If both FEV_1_/FVC and FEV_1_/VC ratios were available, the lowest ratio was used in the evaluation. In addition, FEV_1_%pred was calculated according to the Global Lung Function Initiative (GLI)^36^ in patients having undergone diagnostic spirometry. These FEV_1_%pred values were used to categorise patients in COPD stages 1–4 according to GOLD^1^.

When available, the most recent spirometry during the study period was also retrieved in the same manner as the diagnostic spirometry, in order to assess whether the airway obstruction was persistent over time.

### Statistics

All statistical analyses were performed using IBM SPSS Statistics version 25 (IBM Corporation, Armonk, NY, USA). Descriptive analyses of the baseline study population characteristics were performed using cross-tabulation and the chi-square test. The proportion of performed spirometries, the proportion of spirometries with non-assessable data and the proportion of spirometries with FEV_1_/FVC or FEV_1_/VC ratios over and under 0.70 were calculated and presented as a circle diagram. For comparison, the same procedure was performed on the data from the previous cohort, PRAXIS I, where data was collected between 2000 and 2003. The proportions of performed spirometries and correctly interpreted spirometries were compared between the two cohorts using cross-tabulations and the chi-square test.

Logistic regression was used to analyse associations with non-spirometry verified diagnosis as well as incorrect spirometric diagnosis and several patient-related factors providing odds ratios (OR) for the independent variables. Univariate logistic regression used non-spirometry verified diagnosis of COPD as the dependent variable and patient characteristics as independent variables. In multivariate logistic regression, sex, age and factors with a statistically significant association in the univariate analysis were included. For studying associations with having a diagnosis of COPD in spite of an FEV_1_/FVC or FEV_1_/VC ≥ 0.70, univariate logistic regression was performed in patients with assessable spirometry data, using the same independent variables as in Table 1, with the addition of “change of diagnosis to asthma during the study period”. Multivariate logistic regression was then performed with factors that were statistically significant in the univariate analysis. *P* values < 0.05 were considered statistically significant.

### Ethics

All participants gave written informed consent and returned the signed form together with the patient questionnaire. The study was conducted according to the principals of the Helsinki Declaration. The study was approved by the regional ethical board in Uppsala, Sweden, Dnr 2011/318.

### Reporting summary

Further information on research design is available in the Nature Portfolio Reporting Summary linked to this article.

## Supplementary information

Reporting summary

## Data Availability

Data cannot be made freely available as they are subject to secrecy in accordance with the Swedish Public Access to Information and Secrecy Act, but can be made available to researchers upon request, after approval from the Swedish Ethical Review Authority has been obtained.
