# Supplementary material for: Diagnostic spirometry in COPD is increasing, a comparison of two Swedish cohorts
Source: NPJ Prim Care Respir Med. 2023 Jun 2;33:23. doi: 10.1038/s41533-023-00345-8 (PMC10235108; doi:10.1038/s41533-023-00345-8)
Supplement: Supplementary file 1 — Reporting summary [file 41533_2023_345_MOESM1_ESM.pdf]

nature portfolio

Corresponding author(s): Åsa Athlin

Last updated by author(s): May 10, 2023

Reporting Summary

Nature Portfolio wishes to improve the reproducibility of the work that we publish. This form provides structure for consistency and transparency in reporting. For further information on Nature Portfolio policies, see our Editorial Policies and the Editorial Policy Checklist.

Statistics

For all statistical analyses, confirm that the following items are present in the figure legend, table legend, main text, or Methods section.

n/a	Confirmed	
☐	☒	The exact sample size (*n*) for each experimental group/condition, given as a discrete number and unit of measurement
☐	☒	A statement on whether measurements were taken from distinct samples or whether the same sample was measured repeatedly
☐	☒	The statistical test(s) used AND whether they are one- or two-sided<br>*Only common tests should be described solely by name; describe more complex techniques in the Methods section.*
☐	☒	A description of all covariates tested
☐	☒	A description of any assumptions or corrections, such as tests of normality and adjustment for multiple comparisons
☐	☒	A full description of the statistical parameters including central tendency (e.g. means) or other basic estimates (e.g. regression coefficient) AND variation (e.g. standard deviation) or associated estimates of uncertainty (e.g. confidence intervals)
☐	☒	For null hypothesis testing, the test statistic (e.g. *F*, *t*, *r*) with confidence intervals, effect sizes, degrees of freedom and *P* value noted<br>*Give P values as exact values whenever suitable.*
☒	☐	For Bayesian analysis, information on the choice of priors and Markov chain Monte Carlo settings
☒	☐	For hierarchical and complex designs, identification of the appropriate level for tests and full reporting of outcomes
☒	☐	Estimates of effect sizes (e.g. Cohen's *d*, Pearson's *r*), indicating how they were calculated

Our web collection on statistics for biologists contains articles on many of the points above.

Software and code

Policy information about availability of computer code

Data collection	No software was used
Data analysis	SPSS version 26.0 was used in the statistical analysis

For manuscripts utilizing custom algorithms or software that are central to the research but not yet described in published literature, software must be made available to editors and reviewers. We strongly encourage code deposition in a community repository (e.g. GitHub). See the Nature Portfolio guidelines for submitting code & software for further information.

Data

Policy information about availability of data

All manuscripts must include a data availability statement. This statement should provide the following information, where applicable:

- Accession codes, unique identifiers, or web links for publicly available datasets
- A description of any restrictions on data availability
- For clinical datasets or third party data, please ensure that the statement adheres to our policy

The datasets generated during and/or analysed during the current study are not publicly available as they are subject to secrecy in accordance with the Swedish Public Access to Information and Secrecy Act, but are available from the corresponding author on reasonable request after approval from the Swedish Ethical Review Authority has been obtained.

Human research participants

Policy information about studies involving human research participants and Sex and Gender in Research.

Reporting on sex and gender	The study included both sexes.
Population characteristics	Participants aged 18 to 75 year, (mean 66.9 years SD 6.8) with a doctors´diagnosis of COPD (ICD J44) in their medical records.
Recruitment	Participants were randomly selected from primary and secondary care in central Sweden. All included participants have approved to have their medical redords reviewed by signing a letter of consent.
Ethics oversight	Regional Ethical board in Uppsala, Sweden

Note that full information on the approval of the study protocol must also be provided in the manuscript.

Field-specific reporting

Please select the one below that is the best fit for your research. If you are not sure, read the appropriate sections before making your selection.

☒ Life sciences ☐ Behavioural & social sciences ☐ Ecological, evolutionary & environmental sciences

For a reference copy of the document with all sections, see nature.com/documents/nr-reporting-summary-flat.pdf

Life sciences study design

All studies must disclose on these points even when the disclosure is negative.

Sample size	The sample size was based on a power calculation performed for the comprehensive data collection with the second PRAXIS cohort, using one of the central research questions of exacerbations in patients with COPD. In order to achieve 80% power to demonstrate a significant decrease in exacerbations in with COPD, taking attrition and non-response into account, 2310 patients were invited and 1704 patients included. Of these 703 patients were diagnosed with COPD during the study period, and were herefore included in the present study
Data exclusions	Participants who did not have a date of diagnosis registered
Replication	This was an observational study, no replication was undertaken.
Randomization	This was an observational study, no allocation was necessary.
Blinding	This was an observational study, no blinding was undertaken.

Reporting for specific materials, systems and methods

We require information from authors about some types of materials, experimental systems and methods used in many studies. Here, indicate whether each material, system or method listed is relevant to your study. If you are not sure if a list item applies to your research, read the appropriate section before selecting a response.

Materials & experimental systems

n/a	Involved in the study	
☒	☐	Antibodies
☒	☐	Eukaryotic cell lines
☒	☐	Palaeontology and archaeology
☒	☐	Animals and other organisms
☐	☒	Clinical data
☒	☐	Dual use research of concern

Methods

n/a	Involved in the study	
☒	☐	ChIP-seq
☒	☐	Flow cytometry
☒	☐	MRI-based neuroimaging

Clinical data

Policy information about clinical studies

All manuscripts should comply with the ICMJE guidelines for publication of clinical research and a completed CONSORT checklist must be included with all submissions.

Clinical trial registration	N/A due to cohort study

Study protocol	N/A due to cohort study
Data collection	Patients with a COPD diagnosis (ICD-10 J44) in their medical records 2007-2010 were randomly selected and asked to answer a questionnaire in 2014. Medical records from the years 2004-2014 were reviewed.
Outcomes	Primary outcome is whether a diagnsotic spirometry was performed or not. Secondary outcome is to evaluate if spirometry results were correctly interpreted in accordance to a COPD diagnosis. Data from medical records and questionnaire were analyzed using cross-tabulation and Chi-square test . Associations to the outcome were analyzed with uni-and multivariate regression analysis.
